# P-2296. Risk Factors for Invasive Aspergillus Infections among Lung Transplant Recipients in the Era of COVID-19 Pandemic

**DOI:** 10.1093/ofid/ofae631.2449

**Published:** 2025-01-29

**Authors:** Thomas Moffitt, Sravanthi Nandavaram, Jaime Soria, Thein Myint

**Affiliations:** University of Kentucky - - Lexington, KY, Lexington, Kentucky; Massachusetts General Hospital, Boston, Massachusetts; University of Kentucky, Lexington, Kentucky; University of Kentucky, Lexington, Kentucky

## Abstract

**Background:**

Invasive aspergillus infection is a major cause of mortality among lung transplant recipients (LTRs). The trends of aspergillus infection appear to be increasing after COVID pandemic.

**Figure:**
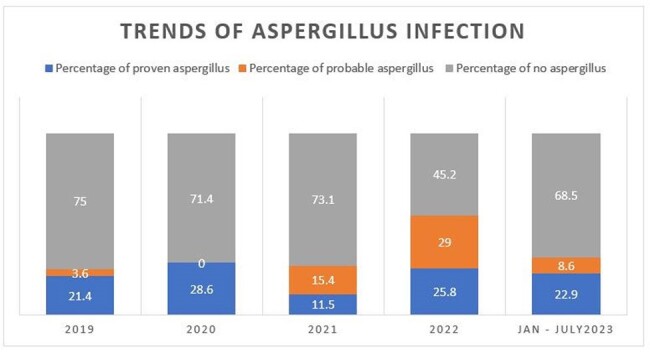

**Methods:**

A retrospective chart review of all LRTs who developed aspergillus infections was performed at our transplant center from January 2019 to July 2023. Proven aspergillus infection is defined as a positive culture or pathology with abnormal imaging, whereas probable aspergillus infection is an elevated aspergillus antigen with abnormal imaging.

**Figure:**
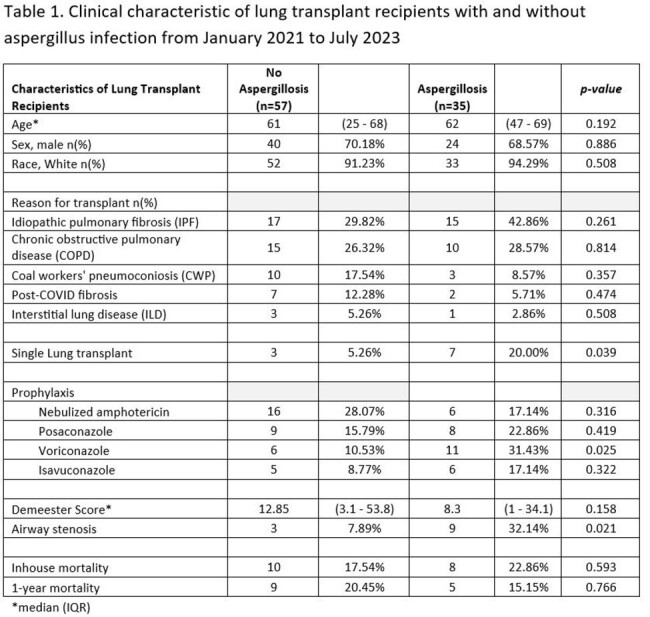

**Results:**

One hundred and thirty-four patients who underwent lung transplantation between January 2019 and July 2023 were followed through December 2023. 21.6% (29/134) and 12.7% (17/134) patients developed proven and probable aspergillus infections, respectively. Although the incidence of proven aspergillus infection has been stable in 2022, the incidence of probable aspergillus infection has increased significantly from 3.6% (1/28) in 2019 to 29% (9/31) (p< 0.01) (Figure 1).

From Jan 2020- July 2023 data analysis, COVID-19 infection was found in 46.2% (18/39) of LTRs diagnosed with aspergillus infection and 45.2% (28/62) of LTRs without diagnosis of aspergillus infection (P=0.92). Of 46 LTRs with COVID-19 infection, 6 (13%) had secondary aspergillus infection.

The clinical characteristics of LTRs with and without invasive aspergillus infection from 2021 to 2023 are shown in Table 1. The risk factors for aspergillus infection include single lung transplant recipients and airway stenosis in multivariate analysis (Table 2). The use of voriconazole was significant for a risk factor in bivariate analysis but not significant in multivariate analysis. Voriconazole prophylaxis was placed for high-risk patients for aspergillus infection.

Inpatient all-cause mortality was 22.86 % in the patients with invasive aspergillus infections compared to 17.54% in LTRs without aspergillosis (p=0.593)

**Table 2:**
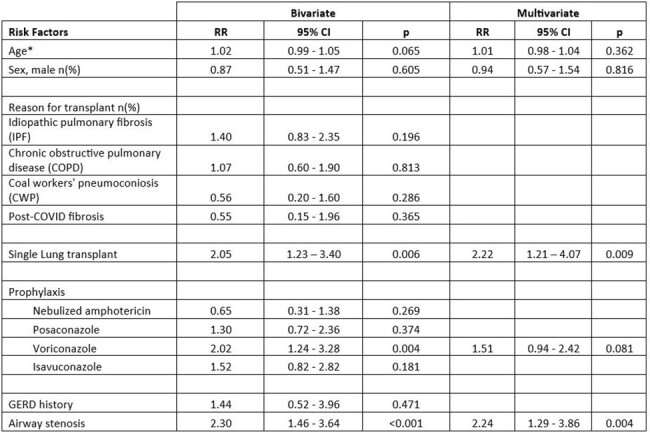
Risk factors for Aspergillus infection among lung transplant recipients

**Conclusion:**

The trends of invasive aspergillus infection in LTRs increased from 25% in 2019 to 54.8% in 2022. COVID-19 infection contributed to a slight increase the incidence of aspergillus infection. The risk factors for invasive aspergillosis include single lung transplant recipients and airway stenosis.

**Disclosures:**

All Authors: No reported disclosures

